# DNA Flow cytometric analysis of the human testicular tissues to investigate the status of spermatogenesis in azoospermic patients

**DOI:** 10.1038/s41598-018-29369-8

**Published:** 2018-07-24

**Authors:** Arka Baksi, S. S. Vasan, Rajan R. Dighe

**Affiliations:** 1 Department of Molecular Reproduction, Development and Genetics, Indian Institute of Science, Bangalore, India; 2 Manipal Fertility, Bangalore, India

## Abstract

A single, rapid and reproducible diagnostic test to predict the type of azoospermia and outcome of sperm retrieval is not yet available. So the feasibility of employing DNA flow cytometry for rapid investigation of the status of spermatogenesis in the patients with azoospermia was investigated. Testicular biopsies of 44 patients with azoospermia undergoing sperm-retrieval surgery and 4 controls were analyzed by flow cytometry to ascertain their testicular germ-cell patterns. The observed germ-cell pattern was further confirmed by RT-PCR analysis of the cell-specific markers and histology for some patients. The patients with Obstructive Azoospermia (OA) exhibited normal spermatogenesis similar to the control fertile patients showing the presence of diploid, double-diploid and haploid cells. The non-obstructive azoospermia (NOA) patients exhibited disrupted spermatogenesis with arrest at the pre-meiotic (only diploid cells present) or meiotic (diploid and double-diploid cells present) stages. The germ-cell pattern, as ascertained by flow cytometry, provided a clear picture of the intra-testicular spermatogenesis and the presence of spermatozoa in the patients’ testes, which was prognostic of their sperm-retrieval. DNA flow cytometry test to ascertain the testicular germ-cell pattern is simple in execution, analysis and interpretation, requires small amount of tissue and provides quantitative data about the status of spermatogenesis in patients. This test would allow comparable analysis of the status of spermatogenesis in patients across clinics and may form the basis for deciding future treatment and intervention strategies.

## Introduction

Azoospermia, a type of male infertility associated with absence of measurable spermatozoa in the semen, accounts for 20–30% of all male infertility cases^1^ affecting about 1% of the male population^2,3^. Sperm retrieval from the testis, followed by Intracytoplasmic sperm injection (ICSI) is one of the procedures adopted for assisted reproduction^4,5^. The common methods for sperm retrieval include testicular sperm aspiration (TESA)^4,6,7^, conventional testicular sperm extraction (TESE)^5,8^ and micro dissection TESE (m-TESE)^9,10^ and in several cases, the patients undergo multiple sperm retrievals^11^. Sperm retrieval rates (SRR) vary depending upon the type of azoospermia with success rates >90% in OA patients which drops sharply to only about 30% in case of NOA^12^. However, the success of sperm retrieval is dependent of the status of spermatogenesis in the testes of the individual and it is absolutely essential to investigate this status in the patients^13^.

Analysis of several pre-operative variables including FSH and LH levels, testicular size and volume have been employed to diagnose the type of azoospermia and predict the outcome of sperm retrieval. However, these factors have low sensitivity and specificity in predicting the success of sperm retrieval^14,15^. A reliable post-intervention predictive method for successful sperm retrieval is histological analysis of the testicular biopsies^16^ which is often the preferred method for diagnosis^17^. This method requires proper fixation of the tissue to enable identification of all the cell types during spermatogenesis. Further, a pathologist should have a thorough understanding of the morphology of individual germ cell types to allow recognition of all cell types^18^. The lack of a systemic approach, divergent reporting systems as well as, the time consuming nature of this analysis (fixation to staining to identification takes several days) often hampers diagnosis and clinical-care^18^. Recent studies have modeled several pre-clinical, non-invasive parameters to try and achieve higher diagnostic accuracy^19–21^, but a single, rapid and reproducible diagnostic test to predict the type of azoospermia (especially in cases of NOA) and outcome of sperm retrieval is not yet available. Thus, there is a need to develop a rapid and simple method of investigating human spermatogenesis.

DNA flow cytometric analyses of the germ cell populations have been reported to accurately and rapidly describe the status of spermatogenesis in the laboratory animal models including primates, and is a quantitative method for analyzing the testicular germ cells^22^. Several groups have also used flow cytometry to show alterations in the testicular germ cell transformations by specific hormonal deprivation or stimulation thereby revealing interesting information on the hormonal regulation of spermatogenesis in rodents and primates^23–26^.

In this study, we explore the feasibility of employing DNA flow cytometry for rapid and accurate investigation of the germ cell status of the individual patients and show that the germ cell pattern accurately reflects the status of spermatogenesis and is predictive of the success of sperm retrieval post-surgery.

## Results

### Profiling of testicular germ cell pattern of individual patients using Flow cytometry

**Figure 1:**
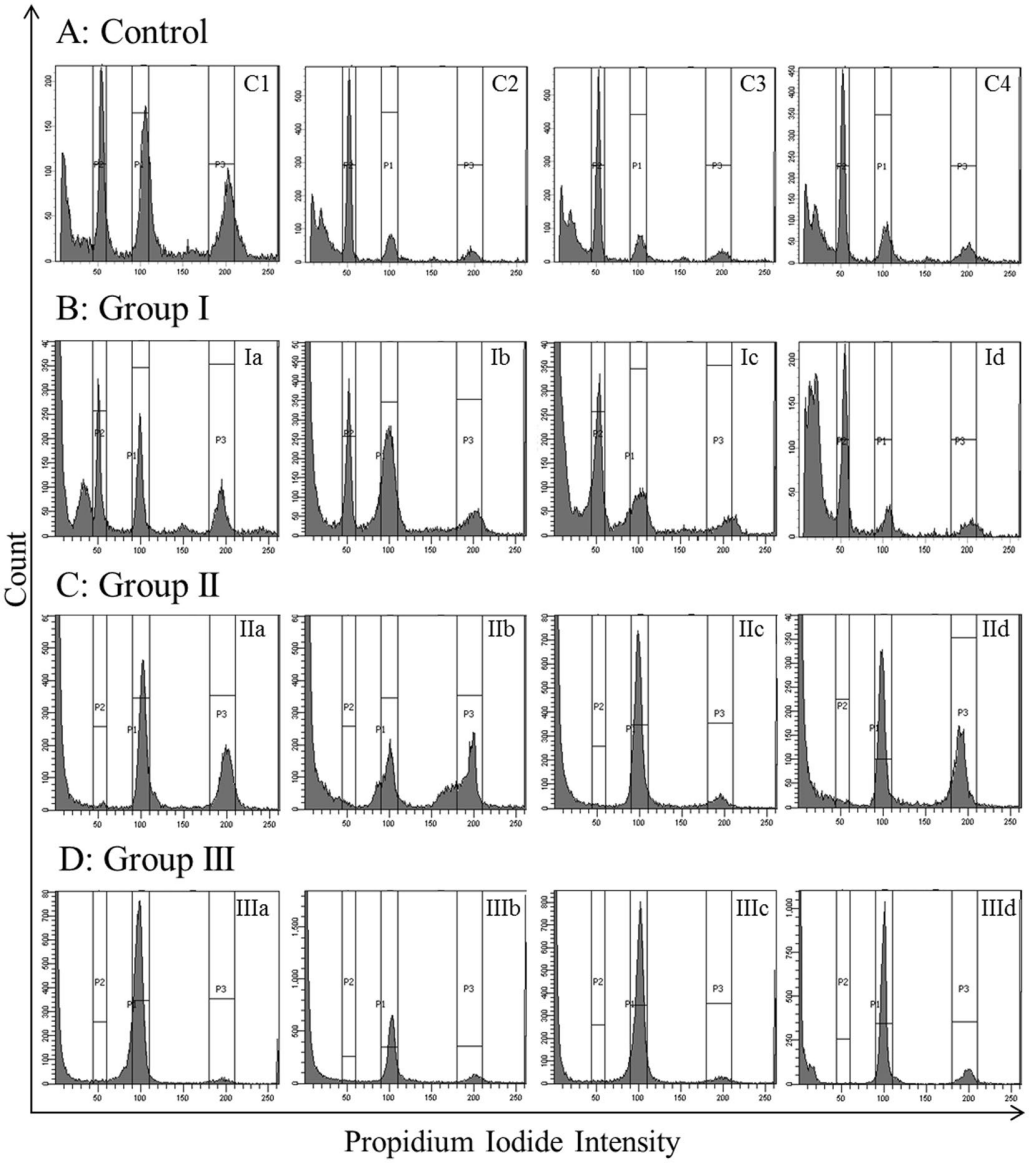
The Germ cell patterns of the control and azoospermic patients. The representative flow cytometric profiles of the testicular biopsy samples as classified into different groups are shown. The testicular tissue samples from the control and azoospermic men were digested with collagenase and the single cell suspensions were fixed with 70% ethanol. The cells stained with PI were analyzed by flow cytometry. Panel A: control- C1-C4; Panel B: Group I – I a-d (obstructive azoospermia, exhibiting haploid, diploid and double-diploid populations); Panel C: Group II – II a-d (NOA with meiotic arrest, exhibiting diploid and double-diploid populations); Panel D: Group III– III a-d (NOA with pre-meiotic arrest, exhibiting diploid populations only).

The testicular tissues of the control and infertile patients were analyzed by flow cytometry and classified according to their cellular patterns (Fig. 1). The germ cell pattern of the control group of patients with proven fertility exhibited three distinct peaks corresponding to the diploid, double-diploid and haploid cell populations. Fourteen patients (Group I), who suffered from OA, also showed three distinct germ cell peaks clearly indicating that spermatogenesis in these patients was complete and equivalent to the control group of patients. The 30 patients suffering from NOA were further divided into two groups based on their flow cytometric profile. The Group II comprising 24 patients showed presence of the diploid and double-diploid cells, but there was complete absence of the haploid cells indicating meiotic arrest of spermatogenesis. The third group of patients (6 patients) showed presence of only the diploid cells with complete absence of the double-diploid and haploid cells indicating the pre-meiotic arrest of spermatogenesis. The characterization of all the human testicular tissue samples is summarized in the Table 1. The clinical diagnosis, the results of flow cytometric tests including percentage of each cell type and the outcome of sperm retrieval are provided in the Supplementary Table I.

**Table 1:** Characterization of testicular tissues of azoospermic patients.

Group	Type of Azoospermia	Number of Samples	Average Age of the patients (years)	Gonadotropin status		Cell Populations present as observed in flow cytometry
				FSH (mIU/ml)	LH (mIU/ml)	
I	Obstructive Azoospermia	14	32.57 ± 3.67	6.56 ± 4.15 CI 95%: 4.04–9.07	6.35 ± 2.79 CI 95%: 4.66–8.04	Diploid, Double-diploid, Haploid
II	Non-Obstructive Azoospermia with meiotic arrest	24	33.21 ± 4.22	10.92 ± 10.10 CI 95%: 5.73–16.12	6.60 ± 3.42 CI 95%: 4.78–8.43	Diploid, Double-diploid
III	Non-Obstructive Azoospermia with pre-meiotic arrest	06	31.17 ± 2.48	22.54 ± 11.36 CI 95%: 12.03–33.06	11.49 ± 8.86 CI 95%: 3.30–19.69	Diploid

Note: All values are shown as mean ± SD.

FSH: Follicle Stimulating Hormone, LH: Luteinizing Hormone, mIU: milli-international units, CI: Confidence Intervals.

To confirm that the flow cytometric profile was a true representation of the testicular germ cell pattern, RT-PCR analysis for the cell population specific markers was performed for each patient. The complete flow cytometric profile of all the patients is provided in Supplementary Figures 1–3.

### Analysis of various gene markers to confirm the germ cell patterns in the patients’ testes

**Figure 2:**
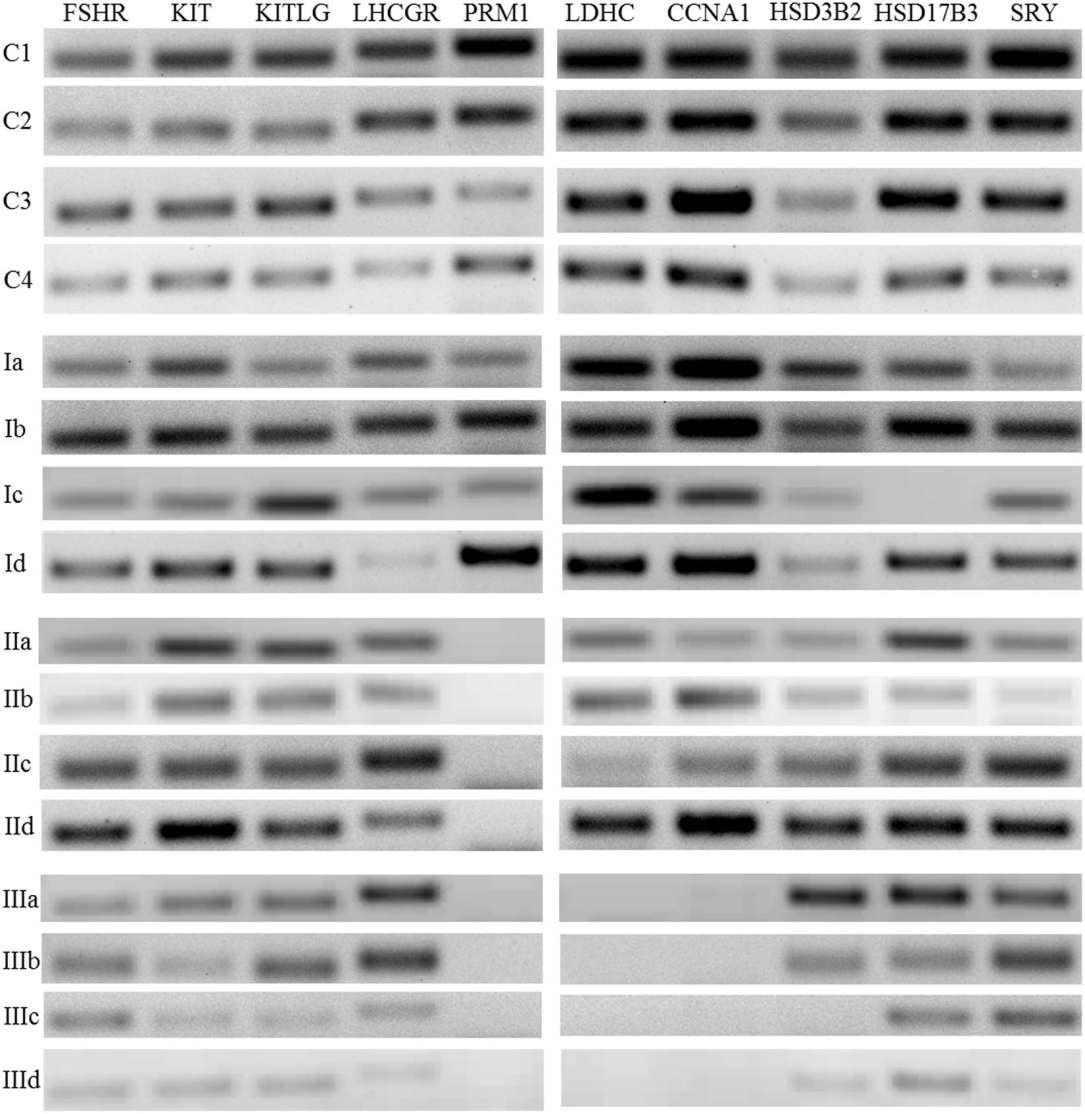
Expression of testicular cell specific marker genes. The expression status of the testicular cell specific markers for each representative patient samples as shown in the Fig. 1 is shown here. The total testicular RNA obtained from tissue samples was converted to cDNA by RT-PCR and the expression of the testicular cell specific markers was analyzed by semi-quantitative PCR using the specific primers. Each patient sample was analyzed individually by PCR for the expression of the testicular cell type specific markers as and when the sample was collected. Cropped images from individual gels are shown. C1–C4: control; I a-d: Group I (obstructive azoospermia, exhibiting haploid, diploid and double-diploid populations); II a-d: Group II - (NOA with meiotic arrest, exhibiting diploid and double-diploid populations); III a-d: Group III (NOA with pre-meiotic arrest, exhibiting diploid populations only).

Various marker genes representative of different cell populations (Sertoli cells, Leydig Cells, Spermatogonia, Spermatocytes/Double-diploid Cells and Spermatids/Haploid cells) found in the human testes were analyzed using semi quantitative PCR. The cell type specific markers chosen are described in the Supplementary Table II. In the control and OA groups of patients, who showed presence of all three germ cell populations, the spermatogonial marker KIT^27^, double-diploid specific markers CCNA1^28^ and LDHC^29^, and the haploid specific marker PRM1^30^ were amplified confirming the presence of three populations of the germ cells. FSHR amplification confirmed the presence of the Sertoli cells while amplification of LHCGR and enzymes of the testosterone biosynthesis pathway HSD3B2 and HSD17B3 confirmed the presence of the Leydig cells^31,32^. The Group II patients did not amplify PRM1, clearly indicating absence of the haploid cells while the Group III patients did not amplify CCNA1, LDHC and PRM1 confirming absence of the double-diploid and haploid cells. However, both Groups showed amplification of FSHR, LHCGR confirming presence of the Sertoli and Leydig cells in patients’ testes. Thus, a clear correlation was seen between the flow cytometric picture of testis and marker gene analysis (Fig. 2).

### Histological analysis of testicular tissues to confirm presence of different germ cells in patients’ testes

**Figure 3:**
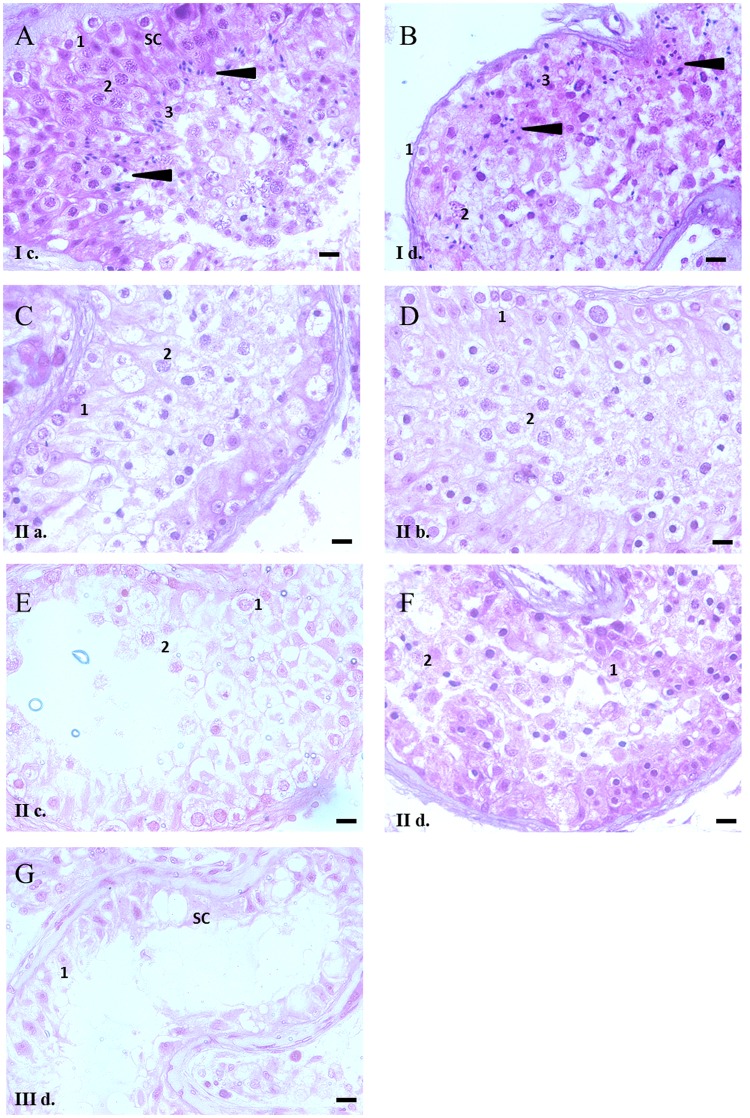
Histological Analysis of tissue samples representative for each group. 5 µm thick testicular tissue sections were stained with Haematoxylin and Eosin and observed under light microscope. The cell types are annotated as SC (the Sertoli cells), 1 (spermatogonia), 2 (spermatocytes) and 3 (round spermatids). Elongated spermatids are marked with black arrowheads. Panels A-B: I c, I d: Group I (obstructive azoospermia, exhibiting haploid, diploid and double-diploid populations); Panels C-F: II a-d: Group II - (NOA with meiotic arrest, exhibiting diploid and double-diploid populations); Panel G: III d: Group III (NOA with pre-meiotic arrest, exhibiting diploid populations only) (Scale bar = 20 µm, Magnification 40X).

Testicular histology of some of the patients in each group was carried out and various cell types were identified as described by McLachlan *et al*.^18^ (Fig. 3). The OA group of patients (Group I) exhibited a typical cellular picture comparable to the normal fertile individuals with presence of all three populations of the germ cells and the normal tubular structure, which is in agreement with the flow cytometric analysis of the testis. All cell types were present in the tubule including the Sertoli cells (SC), spermatogonia, spermatocytes, round spermatids as well as elongated spermatids (Fig. 3A and B). The Group II patients showed complete absence of the haploid cells and the spermatozoa with accumulation of the double-diploid cells in the lumen of the tubules (Fig. 3C–F). In the Group III tissues a clear lack of both haploid and double-diploid cells was observed, with only the Sertoli cells and a few germ cells being present inside the tubule (Fig. 3G). All the different samples examined by histology is shown in Supplementary Figure 4.

### Statistical significance of the flow cytometry test

The percentage of each testicular cell type (haploid, diploid and double-diploid) in each testicular biopsy, as ascertained by flow cytometry, was correlated to the sperm retrieval from each individual patient. As the correlation was drawn between a continuous variable (percentage of cells) and a dichotomous variable (sperm retrieval), the Point Biserial correlation coefficient (R_PB_) was calculated and was observed to be the same as the Pearson correlation coefficient (R_P_). The percentage of the haploid cells showed a positive correlation (R_PB_: +0.89, R_P_: +0.8922, CI 95%: 0.8099 to 0.9401; p < 0.0001) to sperm retrieval, while the percentage of diploid cells showed a negative correlation (R_PB_: −0.78, R_P_: −0.7798, CI95%: −0.8743 to −0.6284; p < 0.0001). The double-diploid cells did not show any correlation to the status of sperm retrieval (R_P B_: +0.05, R_P_: +0.0513, CI 95%: −0.2494 to 0.3430; p = 0.743).

The means of the percentage of the haploid and diploid cells from the patients with positive (M_SR+_) and negative (M_SR-_) sperm retrieval was significantly different. The M_SR+_  ± SEM was 40.86 ± 4.146 and M_SR- _± SEM was 3.869 ± 0.4976 for the haploid cells with p value < 0.0001, while for the diploid cells the M_SR+_  ± SEM was 40.28 ± 4.299 and M_SR- _± SEM was 78.54 ± 2.546 with p value < 0.0001.

The specificity analysis for the flow cytometric test was carried out using the percentage of the haploid cells and the status of sperm retrieval. The specificity and sensitivity was calculated for a range of thresholds of percentage of the haploid cells ranging from 0–70%. The specificity and sensitivity were unchanged in the threshold-range of 13–22% of the haploid cells. Subsequently, the percentage of the haploid cells ≥20% was fixed as the threshold for positive sperm retrieval. In the flow cytometric test, a testicular biopsy sample from a patient diagnosed with OA showed a very low percentage of the haploid cells (10.78%; OA 10), but had successful sperm retrieval. This was annotated as the false negative. The detailed statistical analysis is shown in the Table 2. The significance of the percentage of haploid cells in predicting sperm retrieval was evaluated using Fisher’s exact test. The flow cytometric test showed high sensitivity (~92.86%) and specificity (~100%) (p value < 0.0001) in predicting the outcome of sperm retreival. The false positive rate (α = 1 − specificity) was 0% while the false negative rate (β = 1 – sensitivity) was 7.14%. The binomial proportion for the same was 0.9773 and the CI95% was 0.8798–0.9944. Thus the flow cytometric test is highly specific and sensitive in predicting the the outcome of sperm retrieval in the azoospermic patients.

**Table 2:** Specificity and Sensitivity of the flow cytometric test.

		Percentage of haploid cells		
		High (≥20%)	Low (≤20%)	
Status of sperm retrieval	positive	13 (TP)	1 (FN)	Sensitivity = TP/(TP + FN) = 13/(13 + 1) = 0.92857 (~92.86%)
	negative	0 (FP)	30 (TN)	Specificity = TN/(FP + TN) = 30 (30 + 0) = 1 (~100%)
		Positive predictive value = TP/(TP + FP) = 13/(13 + 0) = 1 (~100%)	Negative predictive value = TN/(FN + TN) = 30 (30 + 1) = 0.96774 (~96.77%)	

Note: TP: true positive, FN: false negative, FP: false positive, TN: true negative.

## Discussion and Conclusion

The results described above clearly show that flow cytometric test can be employed to rapidly establish the status of intra-testicular spermatogenesis in patients with azoospermia, as well as, to predict the exact nature of the arrest in spermatogenesis. The test is highly sensitive and provides a quantitative picture of spermatogenesis using minimal amounts of tissue.

An ideal sperm retrieval method should allow retrieval of sufficient spermatozoa with minimal trauma and with a single intervention. The current retrieval methods such as TESE with multiple biopsies might provide higher SRR, but removal of testicular tissue in large quantities may lead to atrophy^33,34^. TESA or Fine needle aspiration provides a less invasive alternative to TESE, but the rates of sperm retrieval are low as compared to TESE^4,35^. Micro dissection TESE has the highest SRR (almost 17% better than TESE) as compared to the other two techniques^4,36,37^. In all cases, the sperm retrieval is dependent on the status of spermatogenesis within the testis of each individual patient. In this regard, the flow cytometric test as shown in this study provides a clear picture of the intra-testicular spermatogenesis based on which further intervention strategies could be decided. The flow cytometric test performed with small amount of biopsy tissue was sufficient for diagnosis of spermatogenic disruption and the success of sperm retrieval from patients included in the study corroborated with their flow cytometric profile. All patients in the Group I had successful sperm retrieval while it was unsuccessful in the case of patients lacking the haploid cells indicated by their flow cytometric profile, which confirms the efficacy of this method. In cases of repeated retrieval (due to the loss of viability of the cryopreserved sperm upon thawing), success was achieved only if the primary recovery attempt yielded spermatozoa^33,34,38,39^. The present method provides a clear indication of the possibility of presence of the haploid cells and probable sperm retrieval in the first attempt, eliminating the need for multiple retrieval attempts for the patients with NOA. Further, the exact nature of disruption to the extent of early or late meiotic arrest can be ascertained by employing subsequent analysis of the cell type specific markers following flow cytometry. The transcript of LDHC (the testis specific isoform of lactate dehydrogenase) is expressed initially in the leptotene-zygotene spermatocytes cells with maximum expression in the spermatids^29^. As seen in the study, LDHC amplified in 14 of the Group II patients but not in the other 10 patients. This is indicative of the nature of meiotic arrest (early or late) as the expression of LDHC varies in these patients depending on the stage at which the double-diploid cells are arrested. Also, as mentioned above, the nature of pre-meiotic arrest (spermatogonial arrest, SCO) can be further quantified using analysis of marker genes for the different diploid cell populations.

The use of DNA flow cytometry is a rapid and reproducible method to quantify spermatogenesis in different model organisms and humans. The status of spermatogenesis is reflected by presence of three germ cell populations in the flow cytometric analysis^40–42^ and provides a more reliable evaluation than the histological evaluation as the number of cells analyzed is far higher^43^ and provides a quantitative picture of the patient’s spermatogenesis^44–46^. In case of the patient T9 in this study, the double-diploid peak in the flow cytometry was very small that was clearly confirmed by the low number of spermatocytes seen in the histological section while in case of patients T4, T11 and T16, who exhibited broad double-diploid peaks, also showed presence of large number of the spermatocytes (Fig. 3). Interestingly, the spermatozoa retrieved in case of patients OA10, who exhibited small haploid peak and OA13 who exhibited a large number of dead cells, was also very low (data not shown) clearly indicating the advantage of flow cytometric test in predicting the status of spermatogenesis. In some of the patients, a sub-haploid peak was observed in the histograms. This could be attributed to apoptosis of the germ cells as any arrest in spermatogenesis may lead to cellular degradation and death^47,48^. Conversely, these sub-haploid peaks may also be due to the sperm nuclei staining less intensely due to nuclear compaction^22,49^.

The flow cytometry test to predict the outcome of sperm retrieval has a positive predictive value of 100% and the negative predictive value ~96.77% that are highly significant (Table 2). Further, this test can be performed and completed on the day of tissue collections leading to rapid diagnosis compared to the conventional histology which takes several days. A total of 15 samples were subjected to histological analysis and all of them were in perfect agreement with the flow cytometric profile of the respective sample (Supplementary Figure 4). The test is simple in execution, analysis and interpretation providing a rapid and reproducible method for quantifying spermatogenesis and can be performed across the clinics with comparable results. Further, the results of interpretation do not vary depending upon the experience of the analyst. The major limitations is the invasive nature of the technique, but the uncertainty of the endocrine tests to distinguish OA and NOA and the lack of information about recovery of the mature spermatids from the testis via non-invasive methods necessitates testicular biopsies to assess the status of spermatogenesis of an individual^18^. The flow cytometric test, as in case of histology, is perhaps limited by the area of the testis sampled which maybe be overcome by a secondary biopsy to be decided by the clinician. The cost of this analysis is approximately INR 500 equivalent to less than US$10 excluding the cost of the equipment. In conclusion, we propose flow cytometry as a test that would allow a comparable analysis of the status of spermatogenesis across clinics.

## Materials and Methods

### Ethics Statement

The experiments described here were performed after obtaining appropriate clearances from the Institutional Review Board of the Manipal Ankur Fertility Clinic and the human ethics committee of the Indian Institute of Science (HIEC No: 10/10/2015). Informed consent was obtained from all subjects. All experiments were performed in accordance with the relevant guidelines and regulations with prior approval.

### Human patient samples

Testicular tissue biopsy samples were collected from the azoospermic patients undergoing sperm retrieval prior to their surgical procedure at the Manipal Ankur Fertility Clinic, Bangalore, India. The median age of patients was 32 years with the lower and upper hinges being 30 and 35 years respectively. The individual ages are provided in the Supplementary Table I. The characterization of all the human testicular tissue samples is summarized in the Table 1. Four tissue samples were also obtained from the fertile individuals undergoing testicular surgery for causes other than infertility (CA prostrate and orchidectomy for trauma). The tissue samples were collected in Dulbecco’s Phosphate Buffered Saline (DPBS; Life Technologies Corporation, USA) with 0.5% glucose (Sigma Aldrich, USA) and transported on ice to the laboratory. To obtain single cell preparations of the testicular cells, the tissue samples were suspended in Dulbecco’s Modified Eagle’s Medium (DMEM; Life Technologies Corporation, USA) and digested with 0.04% Collagenase (Type IV; Life Technologies Corporation, USA) and 15 µg/ml DNase-I (Sigma Aldrich, USA) at 32 °C in a shaking water-bath for 90 minutes. At the end of the incubation, the cells were centrifuged at 100 × g for 10 min, washed in DPBS and a fraction of cell preparation was fixed in 70% ethyl-alcohol (Merck Specialities Private Ltd. India) for flow cytometric analysis. The rest of the cells were resuspended in TRI Reagent (Sigma-Aldrich, USA) to extract RNA.

### Flow Cytometry Analysis

The ethanol fixed cells were washed twice with chilled DPBS, incubated with 20 µg/ml RNase-A (Sigma-Aldrich, USA) at 37 °C for 2 hours. The cells were stained with Propidium Iodide (PI; Sigma-Aldrich, USA) (100 μg/ml) for 30 minutes on ice and analyzed in BD FACSVerse (Becton Dickinson, USA) on the basis of their DNA content^24^. The Peripheral Blood mononuclear cells (PBMC) isolated from the human blood were used as controls to fix the voltage for the diploid population and all samples were analyzed at this fixed voltage. The doublet population having high PI-area and PI-width was removed before data acquisition by gating on the PI width versus area dot-plot. The closely clustered populations were considered to be the haploid, diploid and double-diploid populations respectively based on the PBMC peak and are shown as interval gates on the histograms. The diploid cell population was fixed at 100 PI fluorescence intensity (peak-P1) and the haploid and double-diploid populations showed peaks at the half (50) (peak-P2) and double (200) (peak-P3) intensity respectively. Approximately 10,000 cells (excluding the dead cells and debris which were identified based on their forward and side scatters as well as low PI staining intensity) were analyzed for each patient sample. The azoospermic individuals exhibited an arrest of spermatogenesis or a mechanical blockage of the ductal systems leading to accumulation of spermatozoa in the epididymis. In many cases, the tubular architecture was compromised and increased cellular degeneration and death is expected. To reflect this observation, the final histograms shown include the dead cells and debris, though the dead cells were gated to allow the acquisition of 10,000 testicular cells.

### cDNA synthesis and semi quantitative PCR analysis

Total RNA was isolated from each patient’s sample with TRI reagent according to the manufacturer’s protocol. RNA (2 μg) was reverse transcribed into cDNA with random primers using the Revert Aid First Strand cDNA Synthesis kit (Thermo Fisher Scientific, USA) according to the manufacturer’s protocol.

For the marker gene analysis, cDNA equivalent to 20 ng of RNA was amplified by PCR using the 2x PCR Master Mix (Thermo Fisher Scientific, USA). Various marker genes representative of different cell populations were analyzed using semi quantitative PCR (35 cycles, saturation analysis) for ascertaining the presence of a particular cell type in the testis. The KIT^27^ was chosen as the spermatogonial marker while CCNA1^50^, which controls G1-S transition^28^ was chosen as the double-diploid specific marker. In addition LDHC, the testis specific isoform^29^, which is expressed initially in the leptotene-zygotene spermatocytes cells with maximum expression in the spermatids^29^ was also used as the double-diploid specific marker. PRM1, which is exclusively expressed in the post meiotic cells^30^ was chosen to confirm the presence of the haploid germ cells. FSHR was used to confirm the presence of the Sertoli cells while LHCGR and the enzymes involved in the testosterone biosynthesis pathway HSD3B2 and HSD17B3 were used to confirm the presence of the Leydig cells^31,32^. The specific primers for each marker gene and amplicon size are listed in the Supplementary Table II. The housekeeping-gene RPL35 was used as the positive control.

### Histology

A portion of the tissue was fixed in Bouin’s Fixative and subsequently dehydrated, embedded in paraffin and sections of 5 μm thickness were prepared and mounted onto glass slides. For histology, the mounted sections were rehydrated by incubating in varying gradients of alcohol (100%, 80%, 70% and 50%) followed by water. The sections were stained with haematoxylin and Eosin according to Nalbandian *et al*.^51^. The sections were again dehydrated, mounted in DPX and images were captured using a Zeiss microscope and processed by Zeiss Axiocam 4.3 software.

### Statistical Analysis

The data were collected independently for 2 variables – percentage of cells with varied DNA content (haploid, diploid and double-diploid) in the testes of infertile patients using flow cytometry and the status of the sperm retrieval from all patients after surgery. The percentage of each cell type in the flow cytometric test was correlated to the status of sperm retrieval by using both Pearson (R_P_) and Point Biserial (R_PB_) correlation coefficients and the difference in the means (mean percentage of cells when sperm retrieval was positive or negative) was ascertained using the Students t-test with Welch’s correction. Further, the flow cytometry test was analyzed for its sensitivity, specificity and predictive value using Fisher’s exact test and the binomial proportion and its confidence interval was also determined.

## Electronic supplementary material

Supplementary Information
