# Supplementary material for: DNA Flow cytometric analysis of the human testicular tissues to investigate the status of spermatogenesis in azoospermic patients
Source: Sci Rep. 2018 Jul 24;8:11117. doi: 10.1038/s41598-018-29369-8 (PMC6057995; doi:10.1038/s41598-018-29369-8)

**Supplementary Information**

**Title:**

**DNA Flow-cytometric analysis of the human testicular tissues to investigate the status of spermatogenesis in azoospermic patients**

**Authors:**

Arka Baksi^1^ (arkabaksi@iisc.ac.in), Vasan SS^2^ (vasan@manipalfertility.com) and Rajan R. Dighe^1*^ (rdighe@alumni.iisc.ac.in)

**Author Affiliations:**

^1^Department of Molecular Reproduction, Development and Genetics, Indian Institute of Science, Bangalore, India

^2^Manipal Fertility, Bangalore, India

^*^ Corresponding Author

| Supplementary Table I. Clinical diagnosis and percentage of each cell type from flow-cytometry test and outcome of sperm retrieval | | | | | | |
| --- | --- | --- | --- | --- | --- | --- |
| **Biopsy Sample** | **Age** | **Clinical diagnosis** | **% of Cells from flow cytometry** | | | **Sperms retrieved** |
|  |  |  | **Haploid** | **Diploid** | **Double-Diploid** |  |
| OA 1 | 36 | Cryptozoospermia (defective sperms) | 32.92 | 51.8 | 15.28 | Yes |
| OA 2 | 33 | Obstructive Azoospermia (OA) | 30.83 | 57.62 | 11.55 | Yes |
| OA 3 | 28 | OA | 37.58 | 28.98 | 33.45 | Yes |
| OA 4 | 27 | OA | 38.6 | 45.96 | 15.44 | Yes |
| OA 5 | 35 | OA | 36.84 | 37.23 | 25.93 | Yes |
| OA 6 | 32 | OA | 56.66 | 30.89 | 12.46 | Yes |
| OA 7 | 41 | OA | 58.85 | 29.21 | 11.94 | Yes |
| OA 8 | 35 | OA | 30.43 | 46.22 | 23.35 | Yes |
| OA 9 | 30 | Congenital bilateral aplasia of the vas deferens (CBAVD) | 48.32 | 24.92 | 26.76 | Yes |
| OA 10 | 31 | OA | 10.78 | 77.93 | 11.30 | Yes |
| OA 11 | 35 | OA | 55.00 | 24.65 | 20.35 | Yes |
| OA 12 | 32 | CBAVD | 68.07 | 17.61 | 14.32 | Yes |
| OA 13 | 32 | OA | 44.59 | 40.43 | 14.98 | Yes |
| OA 14 | 29 | Cryptozoospermia (immature sperms) | 22.51 | 50.46 | 27.03 | Yes |
| T 1 | 33 | Non-OA (NOA) | 4.69 | 76.95 | 18.36 | No |
| T 2 | 33 | NOA | 1.04 | 85.46 | 13.50 | No |
| T 3 | 32 | NOA | 0.89 | 88.91 | 10.19 | No |
| T 4 | 39 | NOA | 3.05 | 57.45 | 39.50 | No |
| T 5 | 32 | NOA | 3.15 | 67.23 | 29.62 | No |
| T 6 | 30 | NOA | 1.91 | 80.38 | 17.71 | No |
| T 7 | 34 | NOA | 3.12 | 82.56 | 14.33 | No |
| T 8 | 29 | NOA | 4.40 | 88.36 | 07.23 | No |
| T 9 | 39 | NOA | 2.43 | 86.73 | 10.84 | No |
| T 10 | 29 | NOA | 2.88 | 80.89 | 16.24 | No |
| T 11 | 27 | NOA | 4.46 | 40.23 | 55.31 | No |
| T 12 | 37 | NOA | 2.94 | 66.93 | 30.13 | No |
| T 13 | 32 | NOA | 1.76 | 73.47 | 24.77 | No |
| T 14 | 34 | NOA | 6.97 | 80.38 | 12.65 | No |
| T 15 | 42 | NOA | 0.66 | 90.71 | 08.63 | No |
| T 16 | 29 | NOA | 3.77 | 54.46 | 41.78 | No |
| T 17 | 31 | NOA | 3.51 | 88.21 | 8.28 | No |
| T 18 | 38 | NOA | 12.80 | 82.91 | 4.29 | No |
| T 19 | 34 | NOA | 6.51 | 83.06 | 10.43 | No |
| T 20 | 41 | NOA | 8.90 | 64.38 | 26.71 | No |
| T 21 | 34 | NOA | 8.24 | 72.93 | 18.83 | No |
| T 22 | 31 | NOA | 6.00 | 80.26 | 13.73 | No |
| T 23 | 27 | NOA | 5.41 | 48.54 | 46.06 | No |
| T 24 | 30 | NOA | 3.54 | 83.94 | 12.52 | No |
| D 1 | 32 | NOA | 2.32 | 92.38 | 5.3 | No |
| D 2 | 29 | NOA | 4.43 | 90.62 | 4.95 | No |
| D 3 | 32 | NOA | 2.15 | 93.53 | 4.32 | No |
| D 4 | 31 | NOA | 1.94 | 91.63 | 6.44 | No |
| D 5 | 28 | NOA | 1.53 | 90.89 | 7.58 | No |
| D 6 | 35 | NOA | 0.67 | 91.94 | 7.39 | No |

Supplementary Table II. List of primers used

| Primers | **5'<-----Sequence----->3'** | Amplicon Size (in bp) | Specific for |
| --- | --- | --- | --- |
| FSHR Forward | ACAGGGTTTTTCTCTGCCAA | 135 | Sertoli Cells |
| FSHR Reverse | GGTCCCCAAATCCTGAAAAT |  |  |
| KIT Forward | CTGGGATTTTCTCTGCGTTC | 133 | Spermatogonia |
| KIT Reverse | GCCCACGCGGACTATTAAGT |  |  |
| KITL Forward | ACACCACTGTTTGTGCTGGA | 131 | Sertoli Cells |
| KITL Reverse | TCCTGCAGATCCCTTCAGTT |  |  |
| LHCGR Forward | GGCCGGTCTCACTCGAC | 148 | Leydig cells |
| LHCGR Reverse | GAGGTTGTCAAAGGCATTAGC |  |  |
| PRM1 Forward | GCCAGGTACAGATGCTGTCGCAG | 153 | Spermatids |
| PRM1 Reverse | TTAGTGTCTTCTCACTCTCGGTCTG |  |  |
| LDHC Forward | TGCATTGGACAAACTGAAGG | 138 | Spermatocyte/  Spermatid |
| LDHC Reverse | CTTGCACCTGCTGTGACAAT |  |  |
| CCNA1 Forward | GGACAGTGCTAGGGCTGCTA | 136 | Spermatocyte |
| CCNA1 Reverse | CCACAGTCAGGGAGTGCTTT |  |  |
| HSD3B2 Forward | GCCTGTTGGTGGAAGAGAAG | 139 | Leydig Cells, Testosterone biosynthesis pathway |
| HSD3B2 Reverse | GGCTCATCCAGAATGTCTCC |  |  |
| HSD17B3 Forward | CCAAAGTCTTTCTTGCGGTC | 145 |  |
| HSD17B3 Reverse | TGGCCTCTAGTTTTTCCAGC |  |  |
| SRY Forward | AAGATGCTGCCGAAGAATTG | 144 | Pan testicular marker |
| SRY Reverse | TAAGTGGCCTAGCTGGTGCT |  |  |
| RPL35 Forward | TCTAAGATCCGAGTCGTCCG | 142 | Control |
| RPL35 Reverse | GCATGGCACGTGTCTTCTTA |  |  |

**SUPPLEMENTARY FIGURE LEGENDS**

**Supplementary Figure 1**

**Complete profile of the group I patient samples.** The germ cell pattern from flow-cytometry and corresponding marker gene amplification status of 14 samples classified as group I (OA) is shown. Each patient sample was analyzed individually by PCR for the expression of the cell specific markers as and when the sample was collected. Cropped images from individual gels are shown. The representative samples shown in Figure 1 and 2 are annotated on the right with the same labels.

**Supplementary Figure 2**

**Complete profile of the group II patient samples.**  The germ cell pattern from flow-cytometry test and corresponding marker gene amplification status of 24 samples classified as group II (NOA with meiotic/tetraploid arrest) is shown. **A**. Samples T1- T12 **B**. T13- T24. Each patient sample was analyzed individually by PCR for the expression of the cell specific markers as and when the sample was collected. Cropped images from individual gels are shown. The representative samples shown in the Figures 1 and 2 are annotated on the right with the same labels.

**Supplementary Figure 3**

**Complete profile of the group III patient samples.** The germ cell pattern from flow-cytometry test and corresponding marker gene amplification status of 6 samples classified as group III (NOA with pre-meiotic/diploid arrest) is shown. Each patient sample was analyzed individually by PCR for the expression of the cell specific markers as and when the sample was collected. Cropped images from individual gels are shown. The representative samples shown in the Figures 1 and 2 are annotated on the right with the same labels.

**Supplementary Figure 4**

**Histological Analysis of the tissue samples and their corresponding flow-cytometric test profiles.**  The testicular tissue (5μm thick sections) were stained with Haematoxylin and Eosin and observed under light microscope. The corresponding flow-cytometric profile is shown in inset for each sample. The samples are labeled as described in Supplementary Figures 1-3.

Supplementary Figure 1


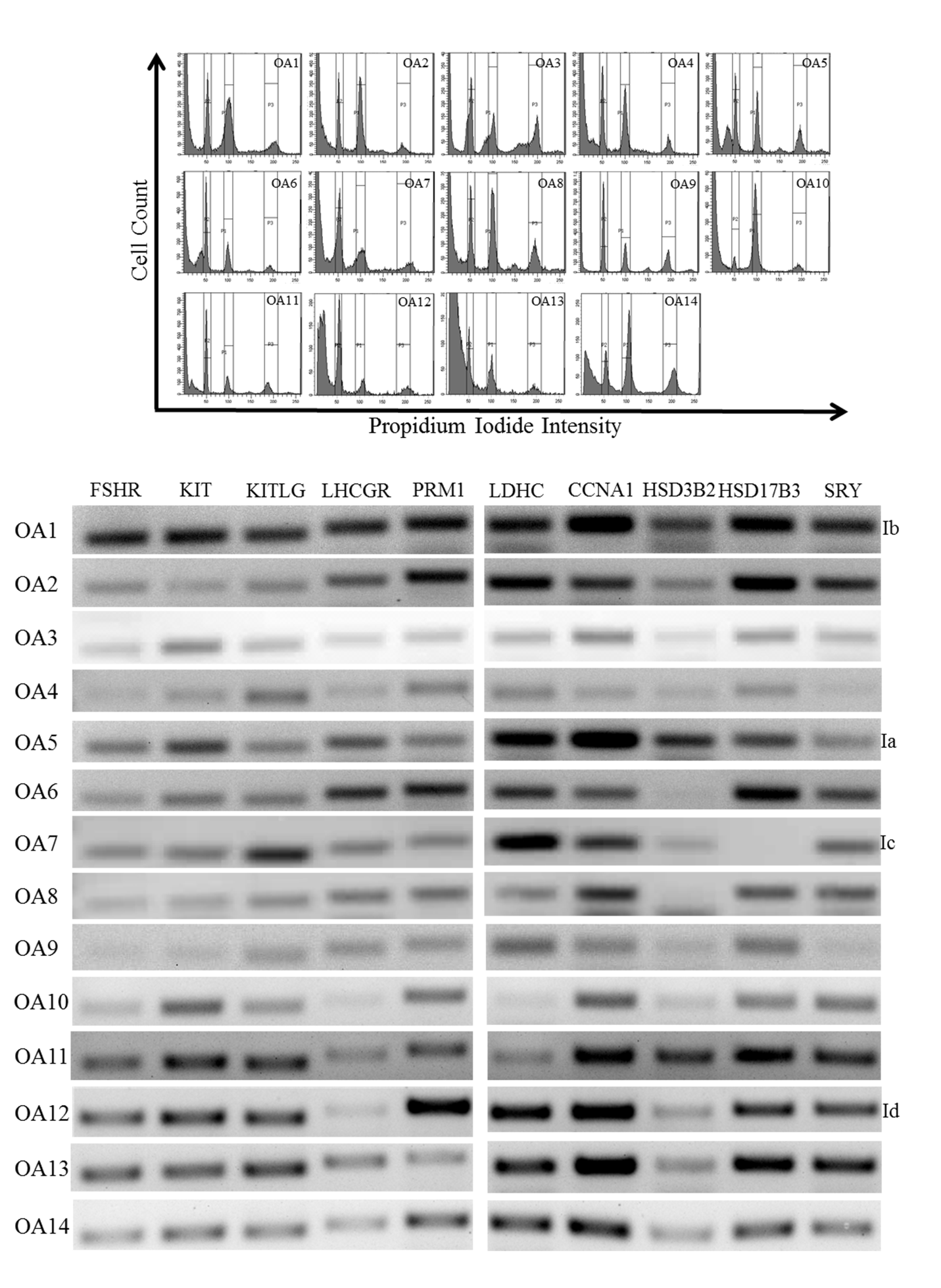


Supplementary Figure 2A


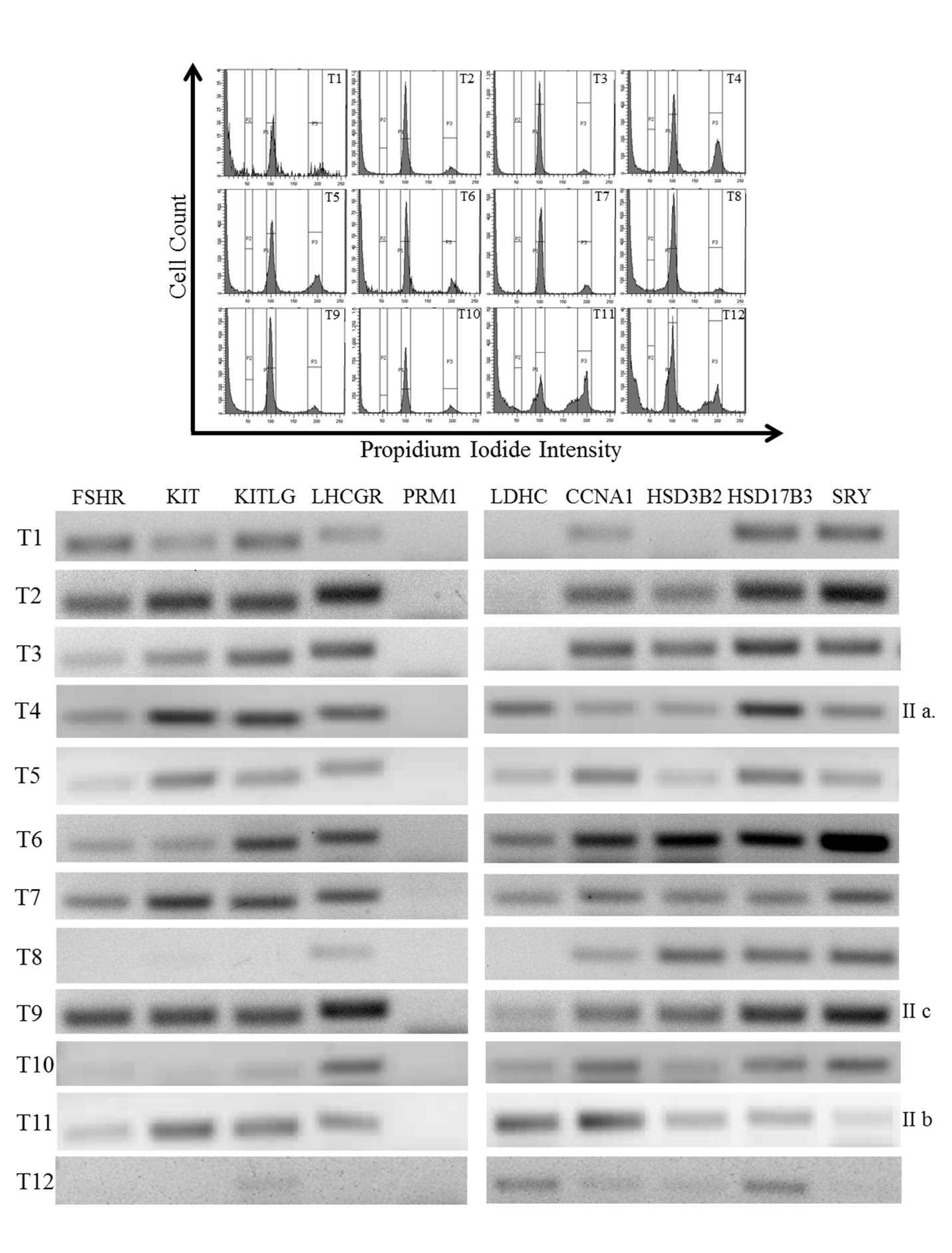


Supplementary Figure 2B


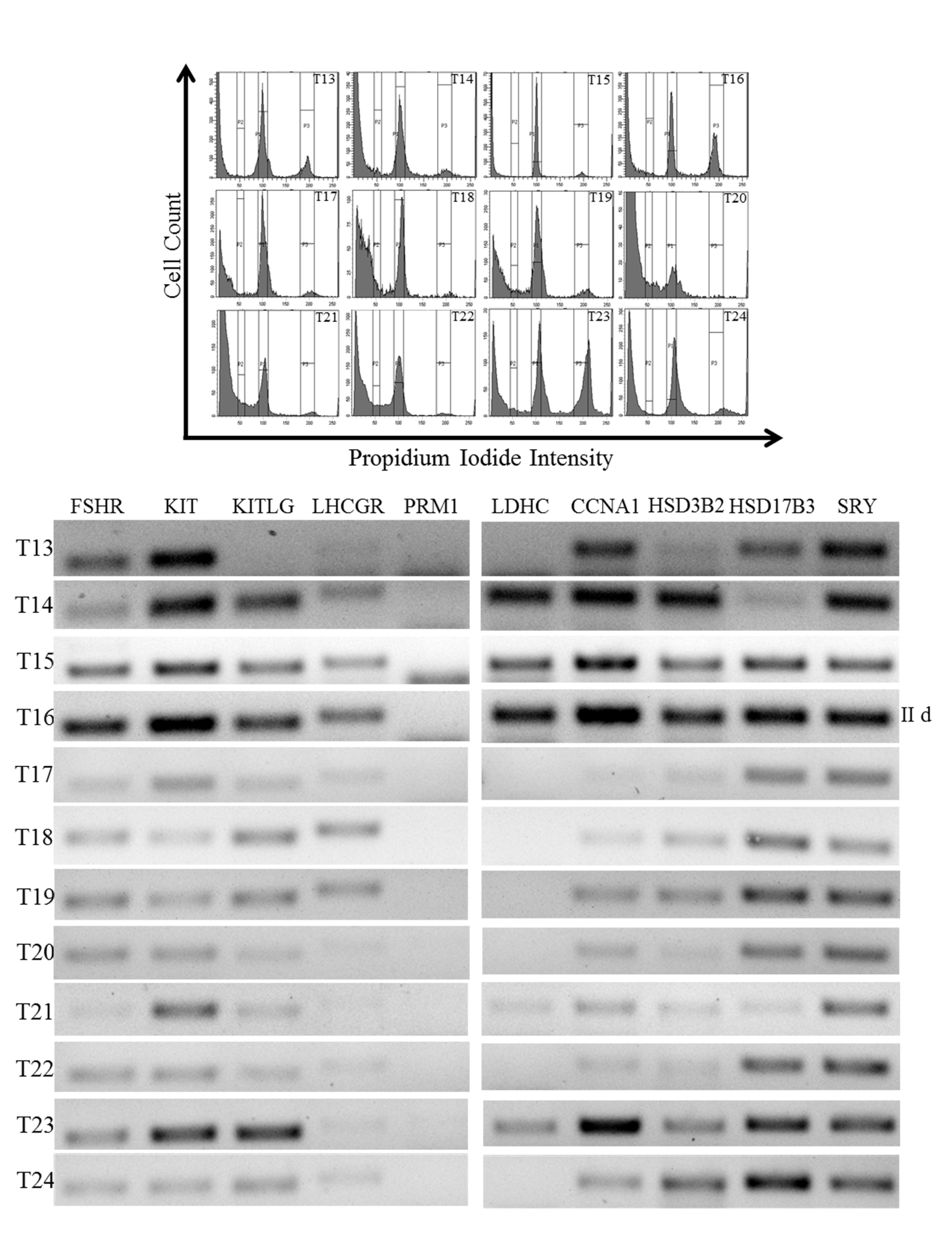


Supplementary Figure 3


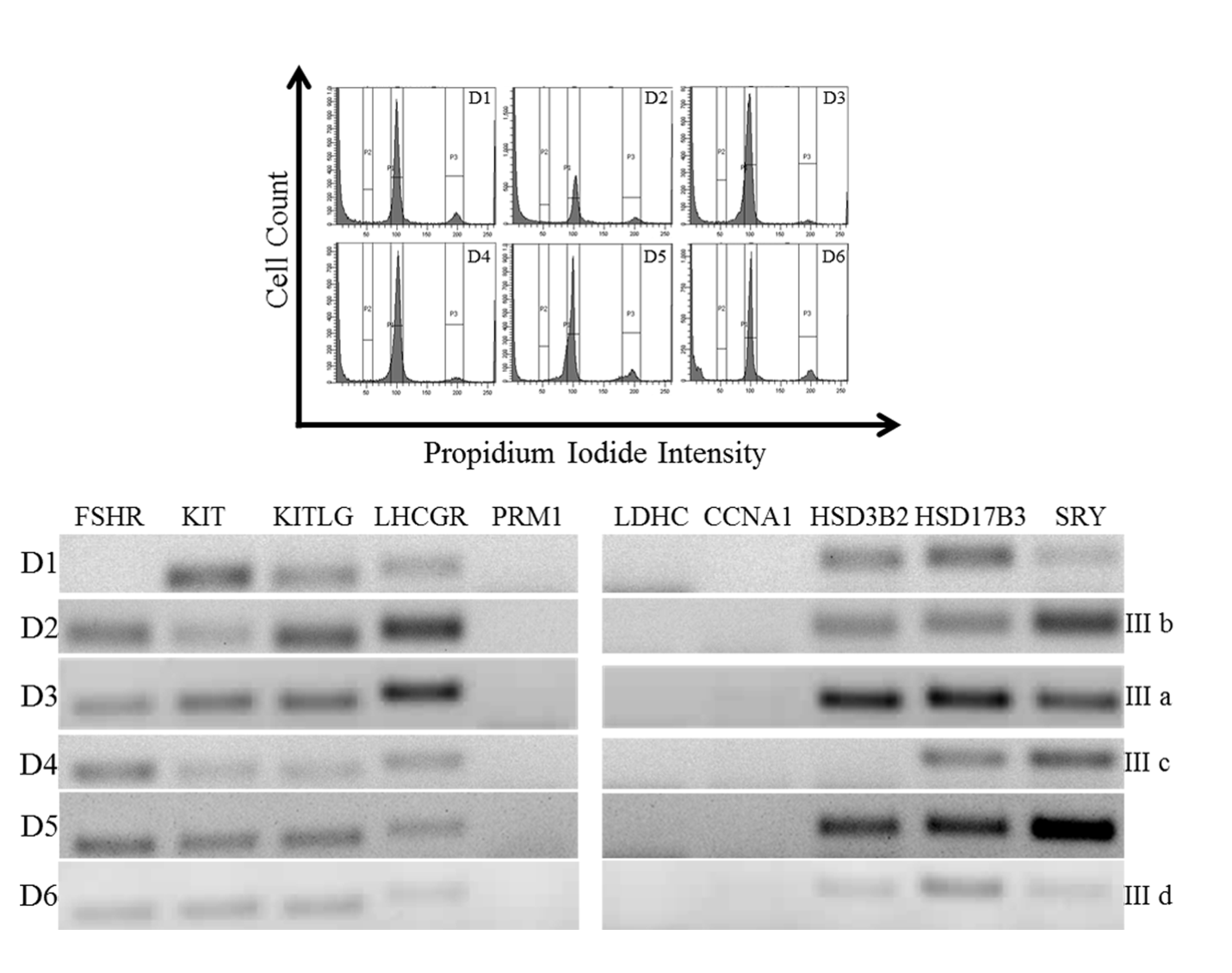


Supplementary Figure 4


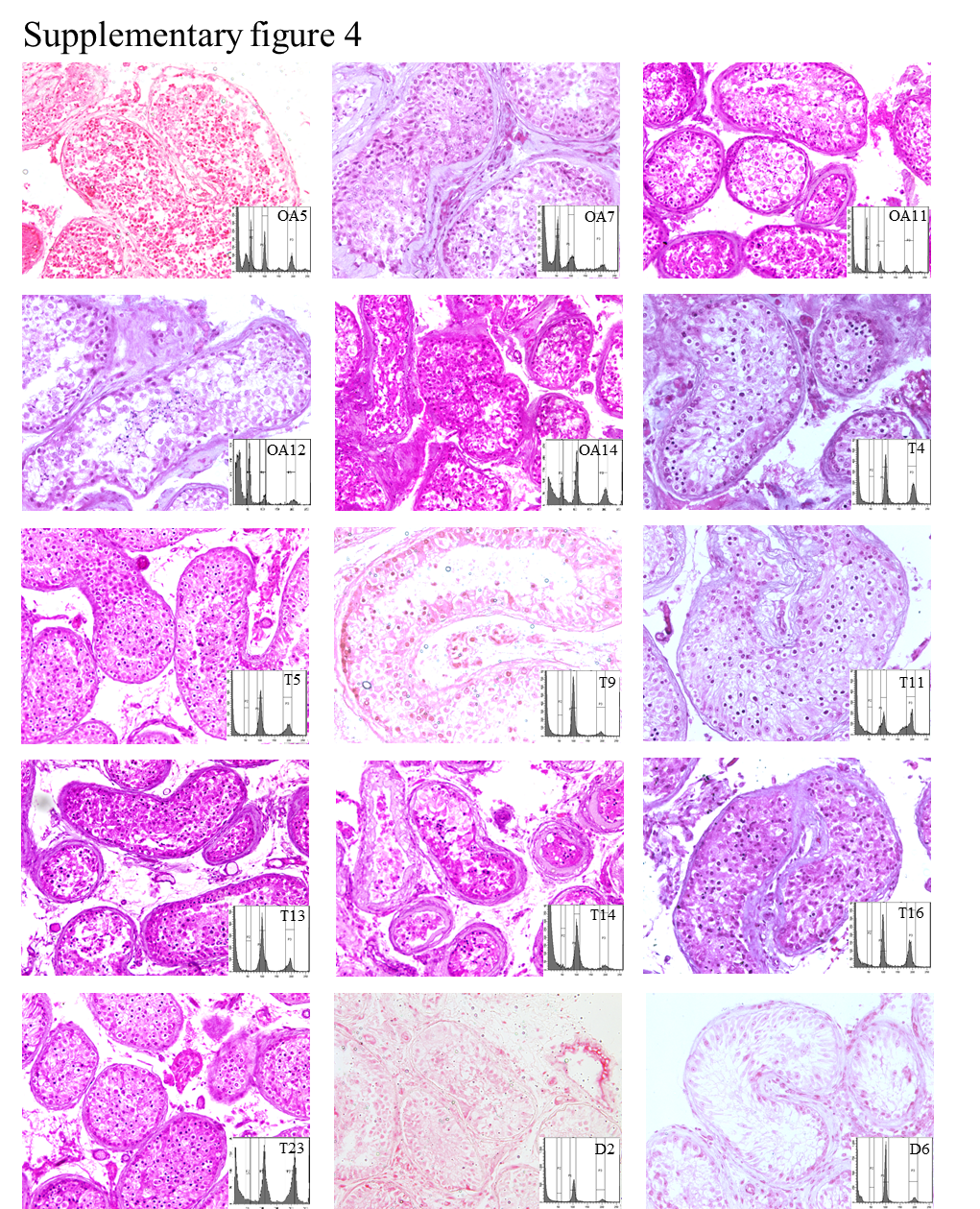

Supplement: Supplementary file 1 — Supplementary Information [file 41598_2018_29369_MOESM1_ESM.docx]
